# Synthesis, Characterization, and Luminescent Properties of Polymer Complexes of Nd(III) with β-Dicarbonyl Ligands

**DOI:** 10.1186/s11671-017-2074-0

**Published:** 2017-05-08

**Authors:** Oleksandra Berezhnytska, Irina Savchenko, Nadiya Ivakha, Olena Trunova, Nataliya Rusakova, Sergiy Smola, Oleksandr Rogovtsov

**Affiliations:** 1 V.I.Vernadsky Institute of General and Inorganic Chemistry, NASU, Kyiv, Ukraine; 2 National Taras Shevchenko University of Kyiv, Kyiv, Ukraine; 3 A.V. Bogatsky Physico-Chemical Institute, NASU, Odessa, Ukraine

**Keywords:** Neodymium(III), Polymer complexes, Spectra, Structure, Luminescence

## Abstract

The neodymium(III) complexes with 2-methyl-5-phenylpenten-1-3,5-dione and allyl-3-oxo-butanoate were synthesized. The polycomplexes on their basis and copolymers with styrene and *N*-vinylcarbazole in ratio 5:95 were obtained by free-radical polymerization. The results of above studies have shown that the configuration of the chelate unit is unchanged during the polymerization. As a result, the type of coordination was determined and the structure of coordination polyhedra was assumed. The luminescence spectra of obtained metallocomplexes and polymers in solutions and solid state are investigated and analyzed.

## Background

Nowadays, metal-containing polymers are of great interest due to a wide range of their practical application in nonlinear optics [1–4] and as sorbents [5], catalysts [6], bactericidal materials [7], etc. Both metal ion and polymeric matrix determine the properties of such copolymers, and it makes it feasible to purposely affect the properties of the final material.

The synthesis and study of lanthanide-containing polymeric compounds based on macromolecular metal chelates is one of the promising areas of coordination chemistry, which is due to the unique properties of both the lanthanides and the metallopolymers on their basis [8]. This class of compounds comprised mainly of the hybrid materials obtained by grafting of lanthanide chelates on the surface of colloidal silica or other silicon-containing compounds and by intercalation of lanthanide salts in the polyvinylcarbazole, polystyrene, or poly(methyl methacrylate) polymeric matrixes [9–13]. Other representatives are the macromolecular metal chelates synthesized by polymerization of monomeric metal complexes [14–16] or by simultaneous polymerization of metal complexes and organic polymers [17, 18]. In our opinion, the use of the latter type of compounds is the most optimal way to obtain functional materials with a uniform distribution of metal in polymeric matrix. In order to implement this approach, a ligand should contain unsaturated double bond making it feasible to use the corresponding metal complex as monomer in polymerization reaction.

The luminescent properties of low-molecular lanthanide complexes [19, 20], hybrid materials on their basis, and lanthanide salts dispersed in polymeric matrixes are investigated rather thoroughly [21–23]. On the other hand, the number of papers dealing with the lanthanide-containing metallopolymers, in which metal ion is chemically bonded with polymeric chain, is considerably less [24–26].

The present work deals with luminescence properties of neodymium(III) complexes with two unsaturated β-dicarbonyl ligands (2-methyl-5-phenyl-1-penten-dione-3,5 (mphpd)) and allyl acetoacetate (allyl-3-oxobutanoate (alacac)) in order to obtain efficient emitting materials. Previously, we have reported only the synthesis and analysis of monomeric complexes with methacroylacetophenone (mphpd) [27].

The main aim of this paper is to study the synthesis of neodymium(III) complexes with alacac and methacroylacetophenone-based copolymers with styrene and *N*-vinylcarbazole and compared the luminescence properties of all compounds obtained.

## Methods

Neodymium(III) nitrate hexahydrate Nd(NO_3_)_3_⋅6H_2_O, allylacetoacetate, styrene, *N*-vinylcarbazole (VK), and other reagents and solvents were of analytical grade. Sodium salt of methacrylacetophenone was synthesized according to the method described in [28]. The result of its chemical analysis and ^1^H NMR study is listed in Table 1.

**Table 1:** Analytical and ^1^H NMR data for Na(mphpd)

Compound	Element: calculated (found), %			^1^H NMR (CDCl_3_), δ (ppm)
	C	H	Na	
C_12_H_11_O_2_Na mphpd	68.55 (68.31)	5.28 (5.20)	10.82 (10.94)	1.98 (s., 3H, CH_**3**_); 3.25 (s., 1H, =C(H)H); 3.62 (s., 1H, =C(H)H); 4.82 (s., 1H, CH); 7.05–7.96 (mult. br., 5H, C_6_H_**5**_)

Synthesis of the Nd(III) complexes with methacrylacetophenone was carried out by the exchange reaction between the equimolar amounts of lanthanide nitrates and Na(mphpd) in a hydroalcoholic solution:

$$ \mathrm{N}\mathrm{d}\;{\left({\mathrm{NO}}_3\right)}_3+3\mathrm{N}\mathrm{a}\left(\mathrm{mphpd}\right)\to \mathrm{N}\mathrm{d}\;{\left(\mathrm{mphpd}\right)}_3+3{\mathrm{NaNO}}_3. $$

Synthesis of Nd(III) complexes with allyl acetoacetate was performed in a hydroalcoholic solution at pH = 8–9:

$$ \mathrm{N}\mathrm{d}{\left({\mathrm{NO}}_3\right)}_3+3\mathrm{H}\left(\mathrm{alacac}\right)\to \mathrm{N}\mathrm{d}{\left(\mathrm{alacac}\right)}_3+{\mathrm{HNO}}_3. $$

The kinetics of polymerization of obtained monomeric complexes was studied by dilatometric method.

A calibrated dilatometer with a volume of 3.594 mL made of heat-resistant glass was put into a water jacket equipped with a UT-15 thermostat. The temperature of heat carrier (distilled water) was controlled with a TL-6 thermometer (measuring accuracy ±0.1 °C). The volume change was measured with a KM-6 cathetometer (measuring accuracy ±0.001 mm). The passed time of the reaction was measured with a stopwatch. The accuracy of time measurement does not exceed 1 s during 30 min. To prepare the solution of monomer, 0.05 mol of monomeric complex, 5 mL of dimethylformamide, and 2,2′-azo-bis(isobutyronitrile) in amount of 1% *w*/*w* were put into a beaker and stirred until complete dissolution. The homogeneous solution thus obtained was transferred into the dilatometer with a long funnel. The dilatometer was attached to a vacuum pump and an argon tank by a three-way stopcock. The cooled dilatometer was firstly vacuumized and then filled with argon. This procedure was carried out three times. Prepared dilatometer filled with argon was sealed, and the reference point was set with the cathetometer at 20.0 °C. Afterwards, the dilatometer was put into the water jacket (the temperature of heat carrier was maintained at 80.0 °C) and the contraction of the solution was monitored with cathetometer. The time reading was started in a few minutes after the initial thermal expansion of liquid had finished. The stopwatch’s readings were taken in 1–5-min increments depending on the rate of contraction. After the polymerization had been completed, the dilatometer was taken out from the water jacket and put into ice-cold water. Its content was quantitatively transferred into a beaker with isopropyl alcohol to precipitate the polymeric complex. The precipitate was filtered off on a glass filter #16 and dried in a vacuum dryer at 40 °C to a constant mass.

**Fig. 1:**
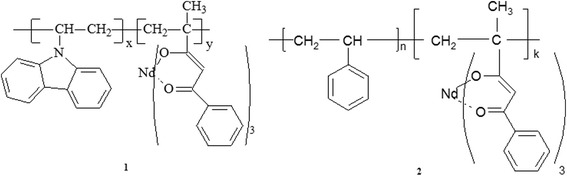
The fragments of copolymers structure (*1*) [Nd(mphpd)_3_]_n_⋅[VK]_m_ and (*2*) [Nd(mphpd)_3_]_n_⋅[styrene]_m_

The polymerization was carried out in 10 wt% dimethyl formamide (DMF) solution of monomers with 2,2′-azobisisobutyronitrile (AIBN) as free radical initiator (1 wt% with respect of monomers mass) at 80 °C for more than 10 h in thermostat. The polymerization mixture was poured into methanol. The solid precipitate was filtered, dissolved in DMF, reprecipitated into methanol, and then dried at 20 °C overnight. Copolymers with styrene and *N*-vinylcarbazole were obtained by free-radical copolymerization in 10 wt% DMF solution of monomers with AIBN as free-radical initiator (1 wt% with respect of monomers mass) at 80 °C for more than 8 h in thermostat. The polymerization mixture was poured into methanol. The solid precipitate was filtered, dissolved in DMF, reprecipitated into methanol, and then dried at 20 °C overnight (Fig.1).

The complexes were studied using a number of physicochemical methods including chemical and thermal analyses, infrared (IR), absorption, luminescent spectroscopies, and dynamic light scattering.

Lanthanide percentage in the complexes was determined using Shimadzu ICPE-9000 atomic emission spectrometer. Thermogravimetry (TG) and differential thermal analysis (DTA) were performed on a Q-1500°D type derivatograph (F. Paulik, J. Paulik, L. Erdey system) in the temperature range from 20 to 800 °C (heating rate 5 °C/min) in a platinum crucible in the presence of anhydrous carrier Al_2_O_3_ in a static air atmosphere. IR spectra were recorded on a Spectrum BX II FT–IR spectrophotometer (Rerkin-Elmer) in the range from 400 to 4000 cm^−1^ in a KBr tablet. Electronic absorption spectra were recorded on Shimadzu UV-1800 spectrophotometer. Particle size determination was performed by dynamic light scattering on a ZetaSizer system (Malvern Instruments). Excitation and luminescence spectra of solid samples and solutions (10^−3^ M in CHCl_3_) were recorded on a Fluorolog FL 3-22 spectrofluorimeter (Horiba Jobin Yvon, 450 W Xe-lamp) using OS 11 filter. DSS-IGA020L InGaAs photoresistor (Electro-Optical Systems, Inc.) cooled to liquid nitrogen temperature was used as a radiation detector for the IR region. Excitation and luminescence spectra were corrected in accordance with the distribution of the radiation sensitivity of xenon lamp and photomultiplier. Powder microphotographs were taken on a Hitachi H-800 scanning electron microscope.

The increase of luminescence efficiency of metallopolymers and copolymers compared with monomers is caused by the absence of water molecules in the nearest coordination environment of the Nd^3+^ ion and an effective sensitization by polymeric matrix [19, 29]. It was established that all complexes are able to show effective luminescence. The luminescence intensity increases in the following series:

$$ \mathrm{N}\mathrm{d}{\left(\mathrm{mphpd}\right)}_3 < \kern0.37em {\left[\mathrm{Nd}{\left(\mathrm{mphpd}\right)}_3\right]}_{\mathrm{n}}\cdot {\left[\mathrm{VK}\right]}_{\mathrm{m}} < \kern0.37em {\left[\mathrm{Nd}{\left(\mathrm{mphpd}\right)}_3\right]}_{\mathrm{n}}\cdot {\left[\mathrm{styrene}\right]}_{\mathrm{m}} < \kern0.37em {\left[\mathrm{Nd}{\left(\mathrm{mphpd}\right)}_3\right]}_{\mathrm{n}}. $$

## Results and Discussion

The kinetics of polymerization of obtained monomeric complexes was studied by dilatometric method, and kinetic parameters of radical polymerization of complexes were calculated: for Nd(alacac)_3_, the rate of polymerization is 1.52°× 10^−4^ mol/l s, the reduced rate of polymerization is 9.39 ×°10^−4^ s^−1^, and the total rate constant is 12.03°× 10^−3^ dm^1.5^/(mol^0.5^ s)^1^, respectively. The kinetic parameters of radical polymerization have very good agreement with literature data that the rate constant is somewhat overstated.

**Fig. 2:**
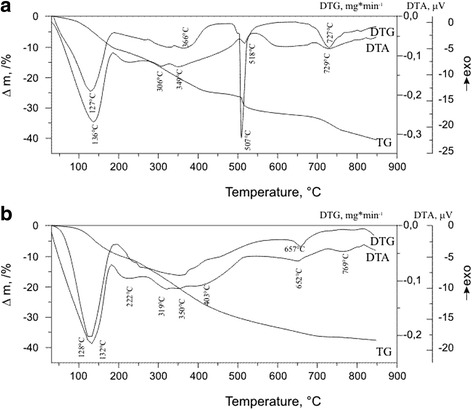
TG, DTG, and DTA curves of Nd(alacac)_3_ (**a**) and [Nd(alacac)_3_]_n_ (**b**)

Thermal analysis of the complexes under study was performed in order to determine their hydrate composition. The decomposition of Nd(alacac)_3_⋅2H_2_O starts at 130 °C with the loss of one adsorbed and two coordinated water molecules (∆*m* = 8.65%, ∆*m*_theor_ = 8.7%). The endothermic effects observed in the 188–562 °C range are due to the gradual loss of three allyl substituents (−CH_2_-CH = CH_2_): the first two detach at 188–487 °C and the third one, in the range of 487–562 °C, the weight loss is 20.9% (∆*m*_theor_ = 20.4%). Further heating accompanied with a number of effects results in the complete decomposition of the organic part of the complex and formation of neodymium oxide (Fig. 2). The total weight loss in the temperature range studied is 40.3%.

The profiles of TG and DTA curves for [Nd(alacac)_3_]_n_ differ from those for the monomeric complex indicating different mechanisms of thermal decomposition. The endothermic effect at 132 °C is caused by dehydration of the metallopolymer, i.e., the loss of water molecules located in its cavities. The weight loss in the 192–602 °C range accompanied with a number of weak endothermic effects is due to both rearrangements and breaking of bonds between adjacent molecules in the polymeric matrix. Unfortunately, it is rather difficult to interpret the thermogram quantitatively since the molecular mass of metallopolymer is unknown. The total weight loss in the temperature range studied is 37.5%. It is notable that for the investigated ligands, there is only one main difference in mechanisms of decomposition: in the next stage after dehydration, in case of methacrylacetophenone complexes, one ligand molecule detaches at temperatures above 260 °C, whereas for allylacetoacetate complexes, the detachment of allyl substituent takes place above 300 °C.

The coordination mode of ligands to lanthanide ions was determined by IR spectroscopy (Fig. 2). The CO and CC stretching vibrations observed in the spectra in the 1500–1600 cm^−1^ region indicate the bidentate-cyclic coordination mode via carbonyl groups (Table 2). The band with the higher frequency (1590–1560 cm^−1^) is attributed mainly to a CO stretching and the band with the lower one (1540–1520 cm^−1^) to a CC stretching [30, 31]. In the spectra of the allyl acetoacetate complexes, the band of CO stretching is shifted towards higher wavenumbers compared to the corresponding methacrylacetophenone complexes. This shift is caused by electron density redistribution in the allyl acetoacetate molecules, and it may indicate the higher stability of the β-ketoesterate chelates compared to the β-diketonates. Weak shoulder at 1640–1650 cm^−1^ is attributed to a stretching of the C=C double bond in ligands. This band almost disappears in the spectra of metallopolymers due to polymerization and presence of only terminal unsaturated groups in them.

**Table 2:** Some distinctive characteristic band of metallic complexes and metallopolymers in the IR spectra

Complex	ν_(M−O)_ + δ_chelate ring_	ν_as_(C–C)	ν_s_(C–O)	ν_s_(C=C)
Nd(mphpd)_3_·2H_2_O	422, 455, 486, 510, 555	1555	1595	1655
[Nd(mphpd)_3_]_n_	418, 438, 458, 480,502, 522, 545,564,587	1556	1604	–
[Nd(mphpd)_3_]_n_[VK]_m_	420,455, 500, 545, 570	1552	1580,1596	–
[Nd(mphpd)_3_]_n_[Styrene]_m_	460, 505, 540,555, 565, 570	1495	1583,1602	1667
Nd(alacac)_3_·2H_2_O	406,415,426, 440, 450, 463, 484, 500, 560	1518,1540	1616,1640	1659
[Nd(alacac)_3_]_n_	420,445,460, 505, 570	1515	1635	–

The broad band of adsorbed and coordinated water molecules presents in the spectra of monomeric complexes in the 3200–3400 cm^−1^ region. The stretching vibrations of Nd–O bonds and chelate rings are observed in the 400–650 cm^−1^ region. It is notable that these bands have different intensity and width in the spectra of monomeric complexes, whereas for metallopolymers, they are split almost equally and have the same intensity. It indicates that all Nd–O bonds are equivalent in metallopolymer molecule and that the polymeric matrix has arranged structure.

The band maxima in the spectra of complexes are slightly shifted towards the long-wave region compared to the spectra of allylacetoacetate and methacrylacetophenone sodium salt (Table 2) indicating the weakening of the metal–ligand bond due to the complexation.

**Fig. 3:**
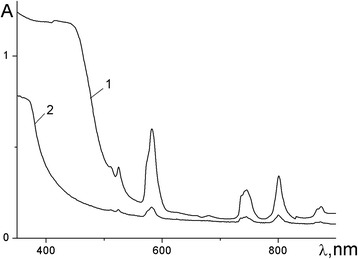
Absorption spectra of (*1*) Nd(mphpd)_3_ and (*2*) [Nd(mphpd)_3_]_n_

The electronic absorption and diffuse reflectance spectra of the neodymium complexes contain a number of bands corresponding to f-f transitions from the ^4^I_9/2_ ground state of the Nd^3+^ ion. From the band splitting, the coordination polyhedron in all complexes is assumed to be a square antiprism. The absorption spectra of metallopolymers are similar to the spectra of monomeric complexes. In the former, the bands are shifted by 10–250 cm^−1^ towards the long-wave region (Table 3) indicating the weakening of the metal–ligand bond in polymers and copolymers. Nevertheless, the profiles of the spectral bands and their splitting remain unchanged, which suggests the same coordination environment of the Nd^3+^ ions both in monomeric and polymeric complexes (Fig. 3).

**Table 3:** Electronic transitions in absorption spectra of neodymium compounds

transition	Nd(mphpd)_3_, cm^−1^	[Nd(mphpd)_3_]_n_, cm^−1^	[Nd(mphpd)_3_]_n_⋅[VK]_m,_ cm^−1^	[Nd(mphpd)_3_]_n_⋅[styrene]_m,_ cm^−1^
^4^I_9/2_ → ^2^P_1/2_	23,255	–	23,150	23,474
^4^I_9/2_ → ^4^G_9/2_	19,530	19,520	19,420	19,880
^4^I_9/2_ → ^4^G_7/2_	19,080	19,040	18,800	–
^4^I_9/2_ → ^2^G_7/2,_^4^G_5/2_	17,215	17,150	17,070	17,240
^4^I_9/2_ → ^2^H_11/2_	15,650	15,480	15,700	15,950
^4^I_9/2_ → ^4^F_9/2_	14,700	14,590	14,540	14,800
^4^I_9/2_ → ^4^F_7/2_	13,420	13,400	13,370	13,460
^4^I_9/2_ → ^4^H_9/2_	12,490	12,400	12,320	12,470
^4^I_9/2_ → ^4^F_3/2_	11,480	11,415	11,440, 11,390	11,495

**Fig. 4:**
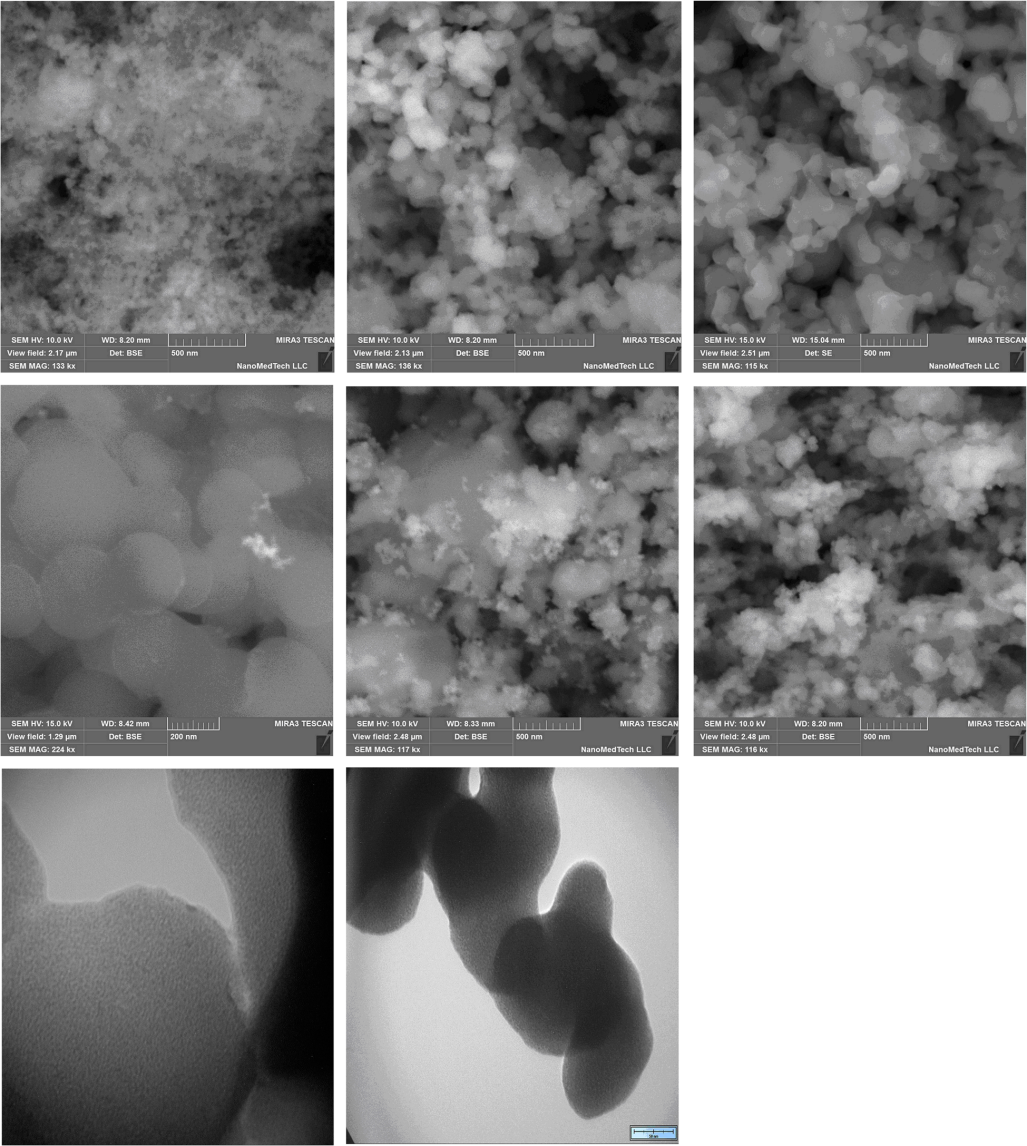
SEM image of powder *1*—Nd(mphpd)_3_, *2*—[Nd(mphpd)_3_]_n_, *3*—[Nd(mphpd)_3_]_n_⋅[VK]_m_, *4*—[Nd(mphpd)_3_]_n_⋅[styrene]_m_, *5*—Nd(alacac)_3_⋅2H_2_O, and *6*—[Nd(alacac)_3_]_n_; TEM image *7*—film [Nd(mphpd)_3_]_n_ and *8*—powder [Nd(mphpd)_3_]_n_

All complexes and metallopolymers were studied by electron microscopy (Fig. 4). Monomeric and polymeric complexes differ significantly. The structure of polymers becomes more ordered, and the successive bonding of monomer molecules into polymeric chains can be seen. The polymeric systems display the formation of net structures with a small agglomeration, which is typical for synthetic polymers. A certain similarity between the metallopolymers based on different ligands can also be noted. The particle size and the degree of agglomeration are greater in the copolymer systems. In the styrene-based copolymer, the globules are much larger in comparison with the *N*-vinylcarbazole copolymer, which is caused by steric factors.

The weakening of the lanthanide–ligand bond in the complexes compared with the aqua-ions and in the metallopolymer compared with monomeric methacrylacetophenone complex allows us to assume that the luminescence efficiency will increase in complexes and metallpolymers.

**Fig. 5:**
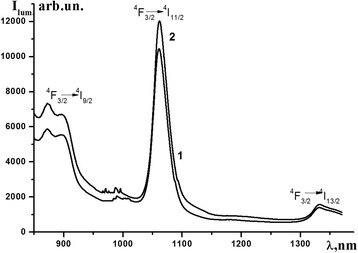
Emission spectra of Nd(alacac)_3_ (*1*) and [Nd(alacac)_3_]_n_ (*2*) in solid state (powder) *T* = 25 °C, *λ* = 362 nm

The excitation spectra of Nd(alacac)_3_⋅2H_2_O and [Nd(alacac)_3_]_n_ in the solid state show a broad band in the 300–400 nm region with a maximum at 355 nm. Under excitation at this maximum, the 4f-luminescence typical for neodymium compounds is observed. The luminescence spectrum of Nd(alacac)_3_⋅2H_2_O (Fig. 5) contains the well-resolved peaks corresponding to the transitions from the excited ^4^F_3/2_ to the ^4^I_9/2_ (872, 877, 892, and 897 nm), ^4^I_11/2_ (1061 nm), and ^4^I_13/2_ (1330 nm) multiplets of ground level of the Nd^3+^ ion. The ^4^F_3/2_ → ^4^I_11/2_ transition band does not split, which indicates a fairly high symmetry of the complex and the presence of only one coordination center. The band of the ^4^F_3/2_ → ^4^I_9/2_ transition splits into four components, which means that the symmetry of the ligand field is not cubic. Under excitation at 355 nm band, the 4f-luminescence which is 1.2 times higher in intensity in comparison with the monomeric complex is observed for [Nd(alacac)_3_]_n_. The band maxima and splitting of the respective transitions (including the most intense ^4^F_3/2_ → ^4^I_11/2_ transition) remain almost unchanged, indicating the same coordination environment of the central ion in both compounds. The quantum yields of the 4f-luminescence are 0.00015 and 0.00018 for monomer and polymer, respectively. Although the luminescence intensity of the allyl acetoacetate complex is higher compared to methacrylacetophenone one [32], the luminescence efficiency is much lower due to the large difference between the energy of the ligand triplet and the Nd^3+^ resonance levels. Solution spectra could not be recorded because no emission bands were observed for the solutions of complexes in chloroform. In this regard, the study of copolymers based on allyl acetoacetate is not reasonable.

The excitation spectra of Nd(mphpd)_3_ and [Nd(mphpd)_3_]_n_ in the solid state show a broad band in the 300–400 nm region with a maximum at 362 nm. The luminescence spectra of these compounds also display three transitions typical for the Nd^3+^ ion: ^4^F_3/2_ → ^4^I_9/2_ (875–902 nm), ^4^F_3/2_ → ^4^I_11/2_ (1061–1062 nm), and ^4^F_3/2_ → ^4^I_13/2_ (1330–1333 nm). The luminescence intensity for the metallopolymer is higher in comparison with the monomeric complex, probably due to the greater number of emitting centers.

The excitation spectrum of copolymer [Nd(mphpd)_3_]_n_⋅[VK]_m_ consists of a broad band in the 300–370 nm region with maximum at about 344 nm. In the case of [Nd(mphpd)_3_]_n_⋅[styrene]_m_, there are two maxima in the excitation spectrum: at 317 nm and 360 nm. These bands are probably originated from π-electron transitions within coordinated ligand systems connected to a polymeric chain.

**Fig. 6:**
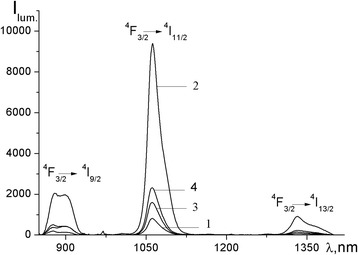
Emission spectra of Nd(mphpd)_3_ (*1*), [Nd(mphpd)_3_]_n_ (*2*), [Nd(mphpd)_3_]_n_⋅[VK]_m_ (*3*), and [Nd(mphpd)_3_]_n_⋅[styrene]_m_ (*4*) in solid state (powder) *λ*_ex_^1,2^ = 362 nm, *λ*_ex_^3,4^ = 350 nm

**Fig. 7:**
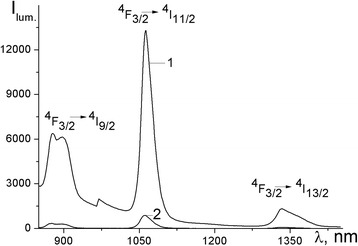
Emission spectra of [Nd(mphpd)_3_]_n_ in solution (*1*) and powder (*2*), *λ*_ex_ =362 nm, *T* = 25 °C

Being excited at the maxima of excitation spectra, the compounds show luminescence in the near IR region typical for neodymium compounds. In the emission spectra, three bands of the 4f-luminescence corresponding to transitions ^4^F_3/2_ → ^4^I_J_ (J = 9/2, 11/2, 13/2) of Nd^3+^ ion are observed (Figs. 6 and 7). The relative contribution of each band in the total integral intensity is 19.5, 69.9, and 10.6% for [Nd(mphpd)_3_]_n_⋅[VK]_m_ and 21.1, 68.5, and 10.4% for [Nd(mphpd)_3_]_n_⋅[styrene]_m_. The ^4^F_3/2_ → ^4^I_9/2_ transition splits into two components: the maxima are at 877 and 899 nm for [Nd(mphpd)_3_]_n_⋅[VK]_m_ and 880 and 899 nm for [Nd(mphpd)_3_]_n_⋅[styrene]_m_. The most intensive band in both copolymers corresponds to the ^4^F_3/2_ → ^4^I_11/2_ transition with the maximum at 1062 nm. The integral intensity of 4f-luminescence of [Nd(mphpd)_3_]_n_⋅[VK]_m_ is almost four times less than [Nd(mphpd)_3_]_n_⋅[styrene]_m_. It was expected that the formation of a copolymer with vinylcarbazole units, which often act as a coordinating ligand [19, 33], will lead to an increase of the Nd^3+^-centered emission. But it was found that the integral intensity of the 4f-luminescence of [Nd(mphpd)_3_]_n_[VK]_m_ is almost four times less than [Nd(mphpd)_3_]_n_[styrene]_m_. This phenomenon can be explained by the fact that vinylcarbazole units remain uncoordinated and do not take part in intramolecular energy transfer processes, since it was determined that the coordination mode of Nd^3+^ ions does not change from monomeric complex to copolymer. Another possible reason is the location of luminescent units in the microcavities of the polystyrene matrix exhibiting more disordered local environments due to the influence of the electric field of the surrounding polystyrene groups. Under this influence, the distortion of the symmetry around the Nd^3+^ ions leads to their polarization, which increases the probability for electronic dipole allowed transitions [34].

## Conclusions

To sum up, in the present work, a series of neodymium complexes and metallopolymers were synthesized and studied in the solid state and in solutions. As a result, the type of coordination was determined and the structure of coordination polyhedra was assumed. It is notable that the structure and symmetry of coordination polyhedra were found to depend only on ligand type and remain unchanged when going from monomer to metallopolymer or copolymer. Thus, the compounds based on β-ketoester allyl acetoacetate have a lower symmetry (C_4_ point group), while the complexes of β-diketone methacrylacetophenone belong to C_4v_ group, which is caused by the nature of ligand and delocalized system of π-bonds in the chelate ring. The metallopolymers display certain orderliness due to the equivalence of bonds and their tendency to self-organization.

## Abbreviations

AIBN: 2,2′-Azobisisobutyronitrile
alacac: Allyl-3-oxobutanoate
DMF: Dimethyl formamide
DTA: Differential thermal analysis
IR: Infrared
Mphpd: 2-Methyl-5-phenyl-1-penten-dione-3,5
SEM: Scanning electron microscope
TG: Thermogravimetry
VK: *N*-Vinylcarbazole

## References

[1] Deun RV, Nockemann P, GoЁrller-Walrand C, Binnemans K (2004). Strong erbium luminescence in the near-infrared telecommunication window. Chem Phys Lett.

[2] Meshhova SB, Topilova ZM, Bolshoy DV, Beltyukova SV, Tsvirko MP, Venchikov VY (1999). Quantum efficiency of the luminescence of ytterbium(III) beta-diketonates. Acta Phys Pol A.

[3] Beeby A, Dickins RS, Faulkner S, Parker D, Williams JAG (1997) Luminescence from ytterbium(III) and its complexes in solution. Chem Commun 15:1401–02

[4] Bünzli J-CG, Eliseeva SV (2010). Lanthanide NIR luminescence for telecommunications, bioanalyses and solar energy conversion. J Rare Erths.

[5] Maspoch D, Ruiz-Molina D, Veciana J (2004). Magnetic nanoporous coordination polymers. J Mat Chem.

[6] Yutkin MP, Dybtzev DM, Fedyn VP (2011). Homochiral porous metal-organic coordination polymers: synthesis, structure and functional properties. Uspehii Chimii.

[7] Shevchenko OV, Zinchenko OY, Voloshanovsky IS, Burenkova KV (2012). Effect of some metal β-diketonates on the bactericidal activity of polymer films against gram-positive microorganisms. Visnyk Odessa Univ Chem.

[8] Aromi G, Gamez P, Reedijk J, Laura EN, Perry MR, Shaver MP (2008). Poly beta-diketones: prime ligands to generate supramolecular metalloclusters. Coord Chem Rew.

[9] Cristovan FH, Nascimento CM, Josef M, Bell V, Laureto E, Duarte JL, Dias IFL, Cruz WO, Marletta A (2006). Synthesis and optical characterization of poly(styrene sulfonate) films doped with Nd(III). Chem Phys.

[10] Hilder M, Junc PC, Lezhnina MM, Warzala M, Kunast UH (2008). Rare earth functionalized polymers. J Alloys Comp.

[11] Ionicheva YuB, Beiharov AG, Lepaev AF, Troitzkii BB, Schelokov RN (1999) Behavior tenoiltriftoratsetonato tris-1,10-phenanthroline-europium in PVC. Ukr khim zhurn 65(5):27–33

[12] Zhao Z, Yu C, Lin L, Hongyan L, Yani H, Xingqiang L, Wai-Kwok W, Richard AJ (2016). Efficient near-infrared (NIR) luminescent PMMA-supported hybrid materials doped with tris-b-diketonate Ln^3+^ complex (Ln = Nd or Yb). J Photochem Photobiol A Chem.

[13] O’Riordan A, O’Connor E, Moynihan S, Nockemann P, Fias P, Van Deun R, Cupertino D, Mackie P, Redmond G (2006). Near infrared electroluminescence from neodymium complex-doped polymer light emitting diodes. Thin Solid Films.

[14] Pomogailo AD (2002). Metal-polymer nanocomposites with controlled molecular architecture. Russ Chem J.

[15] Pomogailo AD, Savostianov VS (1991). Unconventional methods for the synthesis of metal-containing polymers. Uspehii Khimii.

[16] Mazurenko EA, Berezhnytska OS, Zub VYA (2001). Synthesis, structure and β-radical polymerization dyketonatnyh metal complexes with unsaturated substituents. Compoz Polymer Mater.

[17] Pomogailo AD, Savostianov VS (1983). Metal-containing monomers: advances in polymerization and copolymerization. Uspehii Khimii.

[18] Suo Q, Lu F, Shi J, Hong H, Luo J (2009). Studies on synthesis and fluorescent property of rare earth complexes RE(ABMF)_2_AA and copolymers RE(ABMF)_2_AA-co-MMA. J Rare Earths.

[19] Binnemans K (2005). Rare-earth beta-diketonates. Handb Phys Chem Rare Earths.

[20] Vigato PA, Peruzzo V, Tamburini S (2009). The evolution of β-diketone or β-diketophenol ligands and related complexes. Coord Chem Rev.

[21] Görller-Walrand C, Binnemans K (1998) Handbook on the physics and chemistry of rare earth. Elsevier North Holland

[22] Westcotta BL, Seguina TJ, Gruhnb NE (2014). Photoelectron spectroscopy of several lanthanide β-diketonates. J Electron Spectr Rel Phenomena.

[23] Li W, Li J, Li H, Yan P, Hou G, Li G (2014). NIR luminescence of 2-(2,2,2-trifluoroethyl)-1-indone(TFI) neodymium and ytterbium complexes. J Lumin.

[24] Zub VYA, Berezhnitskaya AS, Savchenko IA, Voloshanovskii IS (2004). Synthesis and polymerization of unsaturated β-diketonates of cobalt. Russ J Coordin Chem.

[25] Berezhnytska O, Savchenko I, Denysova Z, Rusakova N, Fedorov YA, Veligura L, Rogovtsov O, Trunova E (2014). The new nanosized system on the basis Eu(III) complexes as precursors for organic electroluminescence diodes. Mol Cryst Liquid Cryst.

[26] Savchenko I, Berezhnytska A, Smola S, Ivakha N (2013). Synthesis and characterization of copolymers of lantanide complexes with styrene. Fr-Ukr J Chem.

[27] Berezhnytska AS, Savchenko IA, Trunova EК, Rogovtsov AA, Ivaha NB (2012) New precursors for nanomaterials based on neodymium complex. DAN NASU 11:132–138

[28] Ufliand IE, Il’chenko IA, Starikov AG, Pomogailo AD. Preraration and reactivity of metal-containing monomers. Transition metal complexes with metakroilatsetofenono Izv AN Ser Khim. 1990:451–53.

[29] Chen P, Shi J, Zhang Y, Wang K, Nie J (2014). EVA film doped with β-diketones macromolecular lanthanide complexes: preparation, characterization and application. Eur Polym J.

[30] Nehoroshkov VP, Kamalov GL, Geltvaiy II (1984). About the relationship between IR spectral properties of ß-diketonates 3d-transition metals and their structure. Russ J Coord Chem.

[31] Nakamoto K. Infrared spectroscopy of inorganic and coordination compounds. – M.: Mir; 1991.

[32] Savchenko I, Bereznytska O, Smola S, Fedorov YA, Ivaha N, Trunova E (2012). Novel polymer metalcomplexes as precursors for electroluminescent materials. Func Mater.

[33] Binnemans K (2009). Lanthanide-based luminescent hybrid materials. Chem Rev.

[34] Zhang X, Wen S, Hu S, Zhang L, Liu L (2010). Electrospinning preparation and luminescence properties of Eu(TTA)_3_Phen/polystyrene composite fibers. J Rare Earths.

